# From molecular signatures to predictive biomarkers: modeling disease pathophysiology and drug mechanism of action

**DOI:** 10.3389/fcell.2014.00037

**Published:** 2014-08-22

**Authors:** Andreas Heinzel, Paul Perco, Gert Mayer, Rainer Oberbauer, Arno Lukas, Bernd Mayer

**Affiliations:** ^1^emergentec biodevelopment GmbHVienna, Austria; ^2^Department of Internal Medicine IV, Medical University of InnsbruckInnsbruck, Austria; ^3^Department of Internal Medicine III, KH Elisabethinen Linz and Medical University of ViennaVienna, Austria

**Keywords:** omics, integration, molecular model, biomarker, target, systems biology, systems pharmacology, precision medicine

## Abstract

Omics profiling significantly expanded the molecular landscape describing clinical phenotypes. Association analysis resulted in first diagnostic and prognostic biomarker signatures entering clinical utility. However, utilizing Omics for deepening our understanding of disease pathophysiology, and further including specific interference with drug mechanism of action on a molecular process level still sees limited added value in the clinical setting. We exemplify a computational workflow for expanding from statistics-based association analysis toward deriving molecular pathway and process models for characterizing phenotypes and drug mechanism of action. Interference analysis on the molecular model level allows identification of predictive biomarker candidates for testing drug response. We discuss this strategy on diabetic nephropathy (DN), a complex clinical phenotype triggered by diabetes and presenting with renal as well as cardiovascular endpoints. A molecular pathway map indicates involvement of multiple molecular mechanisms, and selected biomarker candidates reported as associated with disease progression are identified for specific molecular processes. Selective interference of drug mechanism of action and disease-associated processes is identified for drug classes in clinical use, in turn providing precision medicine hypotheses utilizing predictive biomarkers.

## Introduction

Despite a continuously rising number of clinical trials the rate of bringing novel medication to the clinic is stalling (Pammolli et al., 2011). Here, Omics profiling and high throughput drug screening technologies at the interface of large scale clinical data have triggered novel conceptual strategies aimed at improved patient stratification for enabling precision medicine (Trusheim et al., 2011; Hollebecque et al., 2014). For implementing such approaches a number of issues need to be addressed including: (i) mirroring the clinical categorization of a phenotype on a molecular level description, (ii) spotting molecular factors mechanistically driving disease progression, (iii) drug-based intervention specifically addressing such progression mechanisms, and (iv) predictive biomarkers allowing fit-for-purpose analysis regarding a match of relevant pathophysiology and drug mechanism of action on the individual patient level (Heinzel et al., 2012).

A clinically well-established example is HER2 positive breast cancer characterized by overexpression of a member of the epidermal growth factor receptor family (ERBB2) playing a mechanistic role in progressive disease. In case the factor is proving positive for a patient the specific presentation is amenable for treatment tackling growth signaling (Hicks and Kulkarni, 2008). Still, the clinical presentation of breast cancer shows heterogeneous pathophysiologies apart HER2 positive subtypes. In consequence, when aiming at a comprehensive assessment of progressive breast cancer phenotypes multimarker panels are needed, e.g., implemented by a multiplexed assay holding 70 individual molecular features (Buyse et al., 2006). Such multimarker panels have generally become a promising strategy for characterizing complex clinical presentations, e.g., utilizing a serum marker panel for predicting coronary artery disease in symptomatic patients, or a urinary proteomics profile for early diagnosis of diabetic kidney disease (LaFramboise et al., 2012; Zürbig et al., 2012).

Failure for identifying a single causative factor as proxy for determining progression of a complex clinical phenotype becomes apparent when comparing the performance of marker panels with single markers, with the latter e.g., reviewed by Hellemons et al. for onset and progression of diabetic kidney disease (Hellemons et al., 2012). In clinical practice a different type of biomarker may be utilized, providing a phenotypic readout primarily reflecting the functional status of an organ in contrast to the pathophysiological characteristics. In kidney disease such functional markers are used in patient management as well as clinical trial design, including the estimated glomerular filtration rate (eGFR) and proteinuria (reflecting glomerular filtration and permeation of macromolecules across the glomerular capillary wall, respectively).

Association of these parameters with worsening of diabetic kidney disease, together with increasing incidence of endpoints as cardiovascular events is undisputed (Adler et al., 2003). However, these markers do not provide information on the specific molecular characteristics of the disease. Functional markers render stratification for tailored therapy in the concept of precision medicine essentially impossible.

The molecular pathway of primary interest in the present clinical setting of diabetic kidney disease is the renin-angiotensin system (RAS), in its activity at foremost controlling blood pressure and fluid balance. Blockade of the RAS has been able to reduce the incidence of renal events in patients with and without diabetes mellitus (Ruggenenti et al., 1998; Brenner et al., 2001). In a study by Lewis et al. angiotensin receptor blockade by Irbesartan reduced the risk of a primary composite endpoint (doubling of baseline serum creatinine concentration, development of end-stage renal disease or death from any cause) during a follow up period of 2.6 years by 20% when compared to the placebo (Lewis et al., 2001). Nevertheless, 50% of patients in the Irbesartan group reached the primary endpoint after 54 months. In an effort to increase the efficacy of RAS antagonistic therapy an angiotensin receptor blocker was combined with placebo or the angiotensin converting enzyme (ACE) inhibitor Lisinopril (Fried et al., 2013). The combination therapy did not reduce the incidence of a combined renal endpoint. On the contrary an increased risk of hyperkalemia and acute kidney injury was observed confirming other reports questioning the safety of this approach (Mann et al., 2008; Parving et al., 2008).

Next to addressing RAS, organ-specific molecular processes involving inflammation and oxidative stress have been implicated in progressive tubulointerstitial fibrosis, the best histological, hence molecular mechanistic predictor of an adverse renal disease prognosis (Rodríguez-Iturbe and García García, 2010). Bardoxolone, a nuclear factor-erythroid-2-related factor 2 activator with anti-oxidative capacity increased eGFR in patients with advanced diabetic renal disease (Pergola et al., 2011). However, a large prospective controlled randomized trial with hard endpoints had to be stopped because of severe side effects (De Zeeuw et al., 2013).

As given with these examples for chronic kidney disease (but in its conceptual fundament holding true for a multitude of highly prevalent chronic diseases), many of the recent interventional studies failed to achieve their goals. Here biomarkers promise to take a key role in selecting patients for studies and/or to predict the long term effects of a drug on hard endpoints. Upfront stratification in randomized controlled trials by separating patients by drug response as measured by biomarkers serving as endpoint surrogate and then randomizing the groups separately is an approach which is, at least from a statistical point of view, preferable to *post-hoc* analysis (De Leon, 2012). Such an enrichment strategy is currently e.g., tested in the SONAR study (clinicaltrials.gov reference NCT01858532) addressing diabetic nephropathy (DN).

However, with respect to fit of specific drugs biomarkers need to carry predictive value, i.e., a biomarker shall on a patient-specific level identify responders benefitting from drug effect. In this setting various levels need to be considered involving genetic and environmental components defining disease presentation and progression. The drug target may see genetic polymorphism impacting drug binding, but polymorphism may further involve drug transport and drug metabolism (Johnson, 2001). A significant number of genetic polymorphisms have in the meantime become drug label-relevant regarding drug efficacy, but also toxicity and side effects (U.S. Food and Drug Administration, 2014). Pharmacogenomics has clearly demonstrated that the genetic background of an individual introduces heterogeneity in drug response.

Still, this setting assumes a homogeneous patient population with respect to the molecular mechanistic factors determining disease progression, only exhibiting differences in genetic peculiarities of one and the same molecular mechanistic context. In such setting functional biomarkers appear sufficient for identifying progressive disease, and drug variance is fully explained by the genetic background in regard to the mechanism of action of a specific drug.

A complementary perspective may be that the molecular mechanistic background and progression-relevant molecular factors are *per se* diverse and patient-specific, naturally determining drug response (Mayer et al., 2012). In such scenario a biomarker needs to serve as proxy of key mechanistic factors characterizing and driving a disease on a patient-specific level, combined with educating on the specific interference of disease mechanism with drug mechanism of action. For capturing these constraints a detailed molecular map of a clinical phenotype and its interference with a drug mechanism of action is needed, and here integration of Omics profiling adds to identifying such mechanisms (Fechete et al., 2011; Mühlberger et al., 2012).

An a priori stratification of patients based on an appropriately chosen biomarker panel reflecting the pathophysiology of a given patient (group) allowing to determine a match with a specific drug's mechanism of action appears as promising approach. As recently discussed by Himmelfarb et al. fresh approaches are critical in finding therapies to kidney disease benefiting patients, outlining the importance of improving the translational aspect in clinical research (Himmelfarb and Tuttle, 2013). Here, omics technologies have added significantly to the data landscape characterizing chronic kidney disease, however, in a first instance mainly expanding the candidate set of apparently relevant processes and pathways, going in hand with a large number of biomarker candidates, which individually hamper clinically relevant assessment on disease progression (Fechete et al., 2011; Hellemons et al., 2012).

Integrative approaches in the realm of Systems Biology have been proposed for reaching a consensus description of chronic kidney disease pathophysiology, including molecular models of DN as well as of the reno-cardial axis (He et al., 2012; Komorowsky et al., 2012; Mayer et al., 2012; Heinzel et al., 2013). Still, a translation process needs to be followed, joining disease pathophysiology, stratification markers allowing enrichment strategies, combined with on a molecular mechanistic level matching drugs for allowing precision medicine (Mirnezami et al., 2012). In this work we exemplify such procedure on DN being the major clinical presentation leading to end stage renal disease.

## Materials and methods

### General data sources

Protein coding genes identified as associated with DN were collected from public domain transcriptomics data sources, complemented with molecular features reporting such association in scientific literature. Molecular signatures educating on ACE inhibitor mechanism of action were extracted from public domain transcriptomics sources. Proteins discussed as biomarkers or drug target candidates in the context of DN were extracted from scientific literature, with the set of targets further extended with known drug targets of drugs currently utilized in clinical trials including renal endpoints. Protein-protein interaction information and molecular pathway maps were retrieved from public domain databases.

#### Clinical phenotype molecular data

A literature search in NCBI Pubmed utilizing the query string *diabetic nephropathies[majr] AND (microarray analysis[mh] OR gene expression profiling[mh]) AND humans[mh] NOT review* resulted in 37 transcriptomics studies. Explicitly restricting to explorative, array-based mRNA expression studies on human kidney tissue yielded four studies as suitable for inclusion in further analysis. For Berthier et al. and Cohen et al. expression signatures could be retrieved directly from the publications (Cohen et al., 2008; Berthier et al., 2009). For Woroniecka et al. and Baelde et al. the raw expression profiles were retrieved from Gene Expression Omnibus (GSE30122, GSE1009) (Baelde et al., 2004; Woroniecka et al., 2011). Robust Multi-array Average (RMA) normalization for the data set of Woroniecka et al. and MAS5 normalization for the data set of Baelde et al., followed by Significance Analysis of Microarrays (SAM) was employed for identifying features showing differential regulation comparing diabetic kidney disease and healthy control samples. In case of microdissected sample material separate analysis was done for the glomerular and tubulointerstitial compartment.

To further complement the set of DN-associated features a literature mining approach based on Pubmed Medical Subject Headings (MeSH) annotation and publication to gene links provided in gene2pubmed (ftp://ftp.ncbi.nlm.nih.gov/gene/DATA/gene2pubmed.gz) was executed. A Pubmed search using *diabetic nephropathies[majr] AND human[mh]* as query string was performed for identifying publications of relevance in the context of DN, resulting in 10,766 publications. Protein coding genes explicitly discussed in these publications were extracted from gene2pubmed by filtering based on Pubmed ID and Taxonomy ID (9606 for human).

Finally, the sets of differentially regulated features identified in the individual transcriptomics studies as well as the set of genes from literature extraction were consolidated on the Ensembl gene namespace (Table 1).

**Table 1:** **Diabetic nephropathy molecular data space**.

**Data type**	**Study setup**	**# Protein coding genes**	**References**
Transcriptomics, tissue biopsies	Comparison of healthy references (GFR > 60) and established DN (GFR 30-59);		Berthier et al., 2009
	Glomerular compartment:	5	
	Tubulointerstitial compartment:	7	
Transcriptomics, tissue biopsies	Comparison of healthy references (GFR > 60) and established DN (GFR 30-59);		Woroniecka et al., 2011
	Glomerular compartment:	164	
	Tubulointerstitial compartment:	183	
Transcriptomics, tissue biopsies	Comparison of healthy references (GFR > 60) and patients with type 2 diabetes > 5 years;		Baelde et al., 2004
	Glomerular compartment:	167	
Transcriptomics, tissue biopsies	Comparison of healthy references and established DN (no further details provided)		Cohen et al., 2008
	Tubulointerstitial compartment:	69	
Literature extraction	PubMed MeSH query as defined in main text	415	–
Total number of unique protein coding genes		881	

*Provided is the data type, study setup details, number of protein coding genes identified as DN-associated, and literature reference for a study*.

#### Biomarker and target annotation from scientific literature

A NCBI Pubmed search for publications holding *Diabetic Nephropathies* further qualified by one of the following qualifiers *pathology, physiopathology, enzymology, metabolism, complications, blood, diagnosis, urine*, and *epidemiology* as major MeSH concept, further demanding one of the MeSH concepts *Biological Markers* or *Tumor Markers, Biological* was performed for identifying publications discussing biomarker candidates. For retrieving drug target candidates the term *Diabetic Nephropathies* with the qualifiers *drug therapy* and *therapy* was used, respectively. The search revealed 615 publications for biomarkers and 2,692 for drug targets. Their respective Pubmed IDs were subsequently used for extracting human genes from the gene2pubmed file, resulting in 54 biomarker candidates and 19 drug target candidates.

#### Target annotation via drugs under investigation

Clinical trial data for completed and currently ongoing clinical trials were retrieved from ClinicalTrials.gov (http://clinicaltrials.gov/). The advanced search as provided on the ClinicalTrials.gov webpage was used for identifying studies that fulfilled the following two criteria: *Study Type* equals *Interventional Studies* and *Condition* contains *Diabetic Nephropathy*, revealing 206 clinical studies. Title and trial description were manually reviewed for focus on renal disease, resulting in 124 studies further considered. Respective drug interventions were mapped to their DrugBank entries (Law et al., 2014), extracting human drug targets as listed, being further mapped on the Ensembl gene namespace. In total 86 drug targets were identified using this approach, of which one was also part of the 19 target candidates retrieved from mining of scientific literature essentially covering basic and translational research activities.

#### Drug mechanism of action molecular data

A set of ACE inhibitors was retrieved from the Anatomical Therapeutic Chemical (ATC) classification system maintained by the World Health Organization (WHO). 16 compounds classified under *ACE inhibitors, plain* (ATC code: C09AA) were identified and used for subsequent data extraction from DrugMatrix (https://ntp.niehs.nih.gov/drugmatrix/index.html). For six out of the 16 drugs sets of genes being affected by drug presence in rat kidney tissue after drug administration were available within DrugMatrix. Obtained rat gene sets were subsequently mapped from Unigene IDs (Sayers et al., 2009) to Ensembl rat IDs and from there further to human ortholog genes according to Ensembl (Table 2).

**Table 2:** **Drug mechanism of action data space**.

**Drug name**	**# Protein coding genes**	**Database references**
Benazepril	442	ICX5600735
Captopril	535	ICX5602791
Enalapril	526	ICX5601254
Lisinopril	558	ICX5601689
Quinapril	572	ICX5602295
Ramipril	519	ICX5602317
Total number of unique protein coding genes	2058	

*Given is the drug name, number of associated human protein coding genes identified as significantly affected by drug presence in transcriptomics profiling, and DrugMatrix reference identifier*.

### Molecular pathway and protein interaction data

KEGG and Panther pathway membership information for protein coding genes was obtained via KEGG's REST service and from the plain-text database file available on the Panther web site, respectively (Thomas et al., 2003; Kanehisa et al., 2014). Human protein-protein interaction data from BioGRID, INTACT and Reactome were extracted from the respective plain-text files provided by the individual data sources (Stark et al., 2006; Kerrien et al., 2012; Croft et al., 2014). Gene and protein identifiers provided in the original sources were mapped to their respective Ensembl gene IDs. Protein-protein interaction data were further merged into a protein-protein interaction network using Ensembl gene IDs as common denominator of the individual networks.

### Molecular pathway and process identification

Molecular pathways and processes were analyzed on the one hand on the basis of a literature review of KEGG and Panther pathways already discussed as relevant in the context of DN. In a second approach de-novo identification of DN molecular processes was performed utilizing the DN pathophysiology feature set. A segmentation algorithm for the identification of processes in the DN protein-protein interaction network was pursued for assembling a molecular process model for DN. Utilizing an analogous procedure a molecular mechanism of action model for ACE inhibitors was constructed utilizing expression signatures obtained from DrugMatrix.

#### DN pathways from literature

A NCBI Pubmed search for publications utilizing the query string *“diabetic nephropathy”[ti] OR “diabetic nephropathies”[ti]) AND (pathway[ti] OR pathways[ti])* was performed resulting in 53 publications holding the keywords in the title. Subsequently, named entity recognition was performed to annotate occurrence of pathway names according to KEGG and Panther entries in the title and abstract of these publications. Finally, abstracts holding a pathway name were manually reviewed to ensure an association of the identified pathway in the context of DN, leading to 27 individual pathways discussed in literature as being afflicted with DN. Relations between pathways were inferred based on shared genes and the number of protein-protein interactions spanning across pathway boundaries.

#### Molecular process models

Computing molecular process models followed the procedure described in Mayer et al. (2012); Heinzel et al. (2014). In essence, three main steps are performed: (i) mapping of a feature signature being either the DN pathophysiology association (Table 1) or the ACE mechanism of action set (Table 2) on the consolidated protein interaction network, followed by induced subgraph extraction. Nodes with a degree of zero are removed from the subgraph. (ii) molecular process identification via utilizing a segmentation algorithm (MCODE with default settings, Bader and Hogue, 2003), and (iii) determining inter-process relations defined by the number of protein-protein interactions observed between any actual two molecular processes contrasted against the number of interactions between two random sets of nodes with matching node set size.

#### Enrichment analysis

For identifying significance of enrichment of molecular feature sets in molecular processes and pathways a Fisher's exact test with a significance level set to 0.05 was used. Benjamini Hochberg correction was employed to adjust for multiple testing.

## Results

### DN molecular pathways

**Figure 1:**
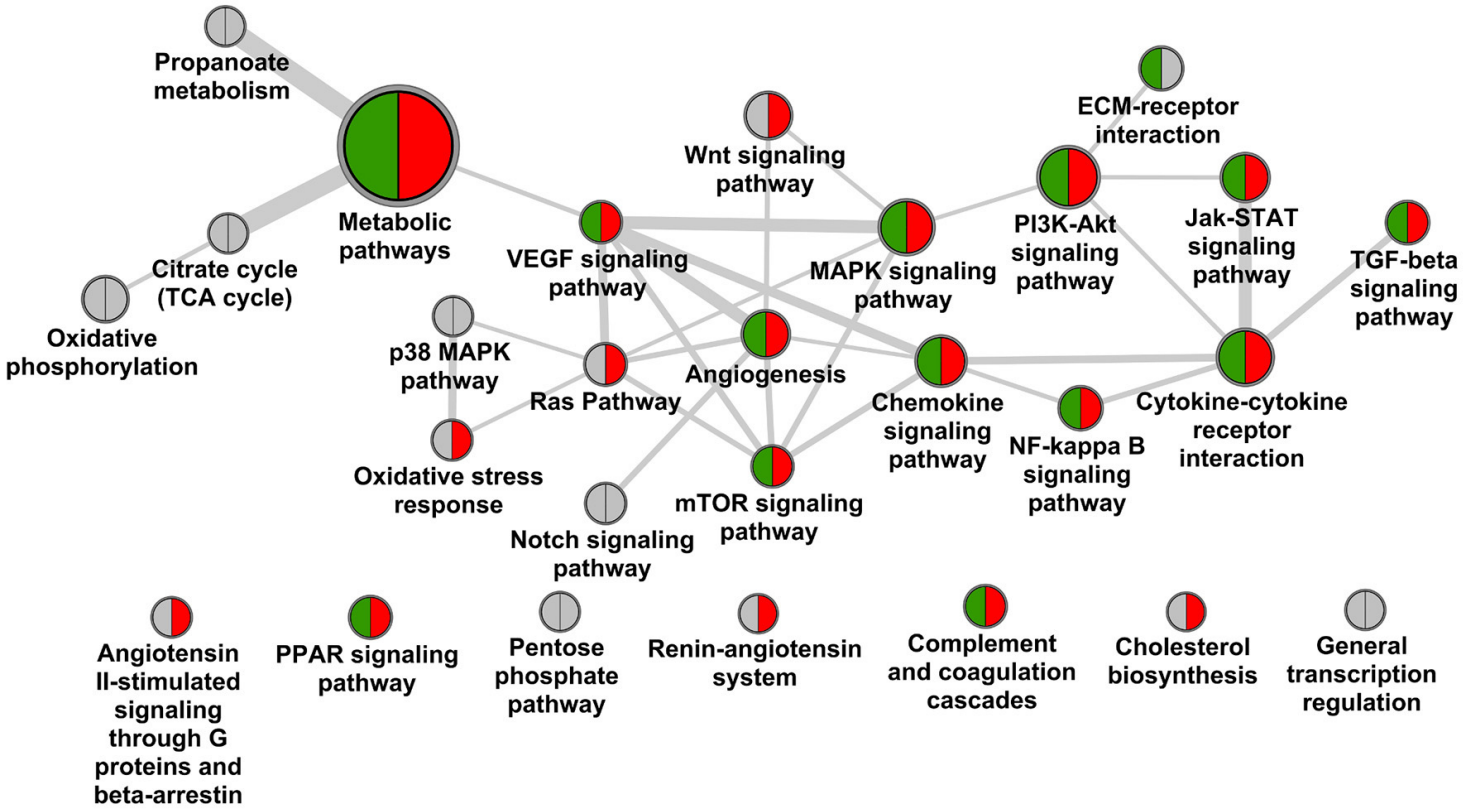
**Pathway landscape of diabetic nephropathy**. Nodes of the graph represent KEGG and Panther pathways (node diameter scales with number of protein coding genes assigned), edges between nodes scale with the number of genes overlapping as well as interactions of genes across pathways according to the protein interaction network. Pathways are marked for holding biomarker candidates (green) and drug target candidates (red).

Screening scientific literature resulted in 27 molecular pathways being observed in the context of DN according to KEGG and Panther pathway annotation (Figure 1). The pathway map is dominated by linked signaling components, with major elements being MAPK-VEGF, and Jak-STAT-cytokine-cytokine receptor interaction further interacting with TGF-beta signaling, covering among others mechanisms of hypoxia response and fibrosis, respectively (Rudnicki et al., 2009; Loeffler and Wolf, 2014). Additional mechanistic aspects include stress response and involvement of extracellular matrix (McLennan et al., 2013; Tan and de Haan, 2014). Further, a number of specific pathways in the context of metabolism are included, as well as the RAS, with the latter however showing no direct links to other pathways on the molecular feature overlap or direct protein interaction level.

Screening for biomarker candidates in scientific literature resulted in 54 protein coding genes, extraction of drug target candidates from literature as well as clinical trials brought forward 104 such genes. Of the 54 biomarker candidates 23 are assigned to the DN pathway map, for the 104 target candidates 52 are involved (Table 3).

**Table 3:** **Molecular pathway annotation, diabetic nephropathy**.

**Pathway name**	**# Genes**	**Biomarker**	**Drug target**	**Enrichment**
Angiogenesis	148	HSPB2, VEGFA, HSPB2-C11orf52	JUN, VEGFA	No
Angiotensin II-stimulated signaling through G proteins and beta-arrestin	35	–	AGTR1	No
Chemokine signaling	190	CCL2, NFKB1, CCL5	CCL2	Yes
Cholesterol biosynthesis	11	–	HMGCR	No
Complement and coagulation cascades	69	F2, FGB, MBL2	SERPIND1, SERPINC1	Yes
Cytokine-cytokine receptor interaction	272	CCL2, LEP, VEGFA, TNFRSF11B, CCL5, PRL, TGFB1	CCL2, TGFB1, VEGFA, TNFSF12, IL18, IL1B, FLT1	Yes
ECM-receptor interaction	87	SPP1, FN1	–	Yes
Jak-STAT signaling	158	LEP, PRL	SOCS1	No
MAPK signaling	256	TGFB1, FGF23, NFKB1	CACNA1H, CACNA1I, CACNB4, CACNA1S, CASP3, CACNA2D3, TGFB1, CACNB3, CACNA1A, CACNA1B, CACNA1C, CACNA1D, CACNA1F, CACNA1G, JUN, CACNB2, CACNG1, IL1B, CACNA2D1, CACNB1	No
Metabolic pathways	1165	XYLT2, PTGDS, KL, PON1, PON2	PTGS2, PDXK, QPRT, ALOX5, NT5E, IMPDH1, ACSL4, XDH, CES1, NNMT, ANPEP, HMGCR, IMPDH2, CYP11B2	No
mTOR signaling	61	VEGFA	PDPK1, VEGFA, INS	No
NF-kappa B signaling	90	NFKB1	PTGS2, IL1B	No
Oxidative stress response	44	–	JUN	No
PI3K-Akt signaling	345	SPP1, VEGFA, FN1, NFKB1, PRL, FGF23	PDPK1, FLT1, VEGFA, INS	Yes
PPAR signaling	71	ADIPOQ	PPARG, ACSL4, FABP1, PDPK1, PPARA, ADIPOQ	No
Ras Pathway	69	–	PDPK1, JUN	No
Renin-angiotensin system	17	–	ACE2, AGTR1, REN, ANPEP, ACE	Yes
TGF-beta signaling	80	TGFB1, SMAD1	TGFB1	No
VEGF signaling	62	VEGFA	PTGS2, VEGFA	No
Wnt signaling	139	–	JUN	Yes
–	–	SPON2, WTAP, UMOD, LCN2, HP, VNN1, AGER, TGFBI, RBP4, NPHS1, HBA1, HBA2, DEFA1B, LPA, CST3, CTGF, ACTA1, PGC, S100A9, DPP4, ALB, CCKAR, GSTP1, DEFA3, S100A8, DEFA1, MMP9, CDH1, S100A4, NPPB, HAVCR1	SOAT1, SLC6A4, ADORA1, MC2R, SIRT1, CYCS, RETN, EDNRA, CRH, EDNRB, KCNA1, ADORA2A, CALM2, CALM3, CALM1, PTX3, PDE3A, KCNMA1, P2RY12, SLC12A1, SLC12A3, GLP1R, DPP4, PDE5A, NR3C2, KCNJ11, ITGB2, KIF6, MMP9, CA12, TUBB1, NAMPT, HCAR3, HCAR2, AR, HBA1, HBA2, CA9, KCNH2, CA2, CA1, CASP1, TUBB, CA4, AHR, CTGF, ABCA1, PDE4A, PDE4B, SCN5A, MMP2, NPC1L1	
Citrate cycle (TCA cycle)	31	–	–	No
General transcription regulation	30	–	–	No
Notch signaling	48	–	–	No
Oxidative phosphorylation	122	–	–	No
p38 MAPK	34	–	–	No
Pentose phosphate	27	–	–	No
Propanoate metabolism	32	–	–	No

*Provided is the KEGG pathway name, number of genes assigned to the pathway according to the pathway source, biomarker, and drug target candidates included in the pathway (gene symbols), and indication of significance of enrichment of such pathway on the basis of the consolidated DN kidney tissue transcriptomics data*.

Significant coverage regarding biomarker as well as target candidates is again seen for central signaling components including chemokine signaling, cytokine-cytokine receptor interaction, complemented by MAPK and PI3K-Akt signaling. Also mechanisms are addressed including key features as VEGFA and TGFB1. No specific targeting is seen for counteracting structural changes in ECM, and minor efforts appear to be assigned to adapting stress response. For seven out of 20 pathways discussed no biomarker or target annotation is identified, and complementary a large number of such features are assigned also outside the pathway landscape presented in Figure 1. Prominent examples for void biomarker assignment include connective tissue growth factor (CTGF) as factor in fibrosis not being assigned in KEGG, the same being true for uromodulin (UMOD) shown to be associated with progressive disease including genetic polymorphisms (Deshmukh et al., 2013; James et al., 2013). CTGF is also discussed in the therapeutic context via utilizing a monoclonal antibody-based approach (Adler et al., 2010).

Testing the DN pathophysiology feature set retrieved from consolidation of transcriptomics profiles regarding enrichment in the given DN pathway landscape identified seven such pathways as significant, however, missing central mechanisms as hypoxia response or TGFB signaling. In contrast other pathways beyond the map given in Figure 1 appeared significantly enriched, including focal adhesion, cell adhesion molecules and adherence junctions, linking to the signaling aspects involved in the disease.

### DN molecular model

**Figure 2:**
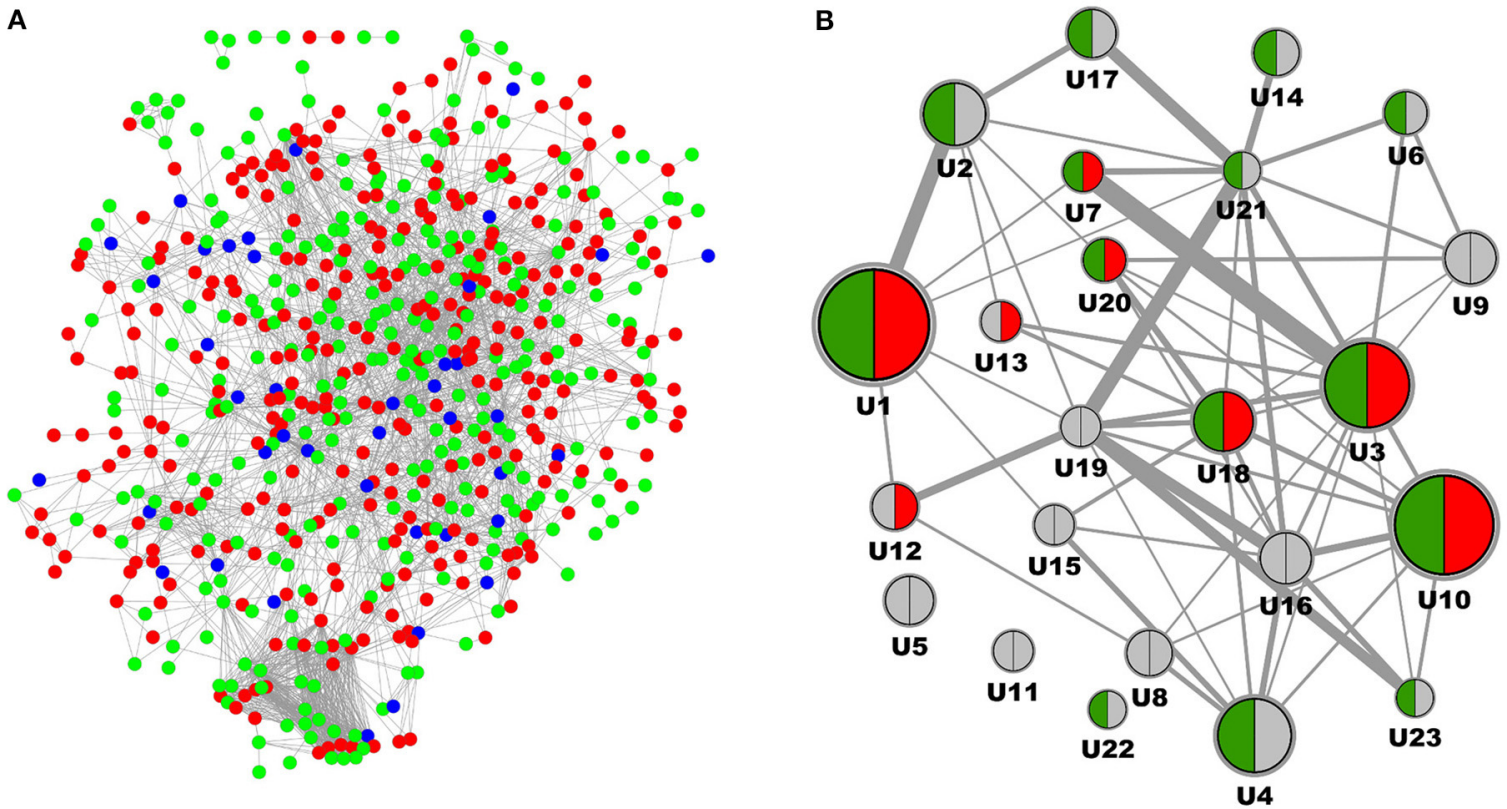
**Molecular model representation of diabetic nephropathy. (A)** Induced subgraph where each node represents a protein coding gene being reported as associated with DN, edges denote interactions according to the underlying interaction network. Features derived from Omics studies are given in red, features delineated from literature mining are given in green, features identified in both data sources are depicted in blue. **(B)** Molecular model representation of DN where each node represents a process segment with the node diameter scaling with the number of protein coding genes involved, and edges between nodes scaling with the number of interactions of genes across nodes according to the protein interaction network. Segments are indicated for holding biomarker candidates (green) and drug target candidates (red).

Complementary to analysis on molecular pathways as defined in KEGG and Panther we performed a network segmentation procedure aimed at identifying DN molecular process segments defined by topological characteristics of the DN-specific subgraph. From the in total 881 protein coding genes included in the DN molecular pathophysiology gene set (Table 1) 880 were also part of the consolidated interaction network, and 634 were identified as member of the induced subgraph (Figure 2A). From the total set of 880 features 246 protein coding genes had no interaction to any other feature of the DN consensus set, hence being disregarded in molecular model computation. Apparent is the relatively minor overlap of features extracted from literature when compared to signatures from transcriptomics. From the in total 516 unique features consolidated from four transcriptomics profiling experiments and 414 features derived from scientific literature 49 are shared.

After MCODE segmentation 200 molecular features remained in process segments, forming a molecular model holding 23 process segments (Figure 2B). Median number of protein coding genes per process segment is 6, with the largest segment encoding 29 features, the smallest 3. Equivalently to the pathway graph in Figure 1 a process graph serves as approximation of individual molecular process characteristics together with their dependencies. Six process segments of the process model hold both, biomarker as well as target candidate annotation, with others encoding just one of the two or none. Of the 54 biomarker candidates 22 are included in the molecular model, the respective number for the 104 targets candidates is 16.

### DN molecular model and drug mechanism of action model interference

**Figure 3:**
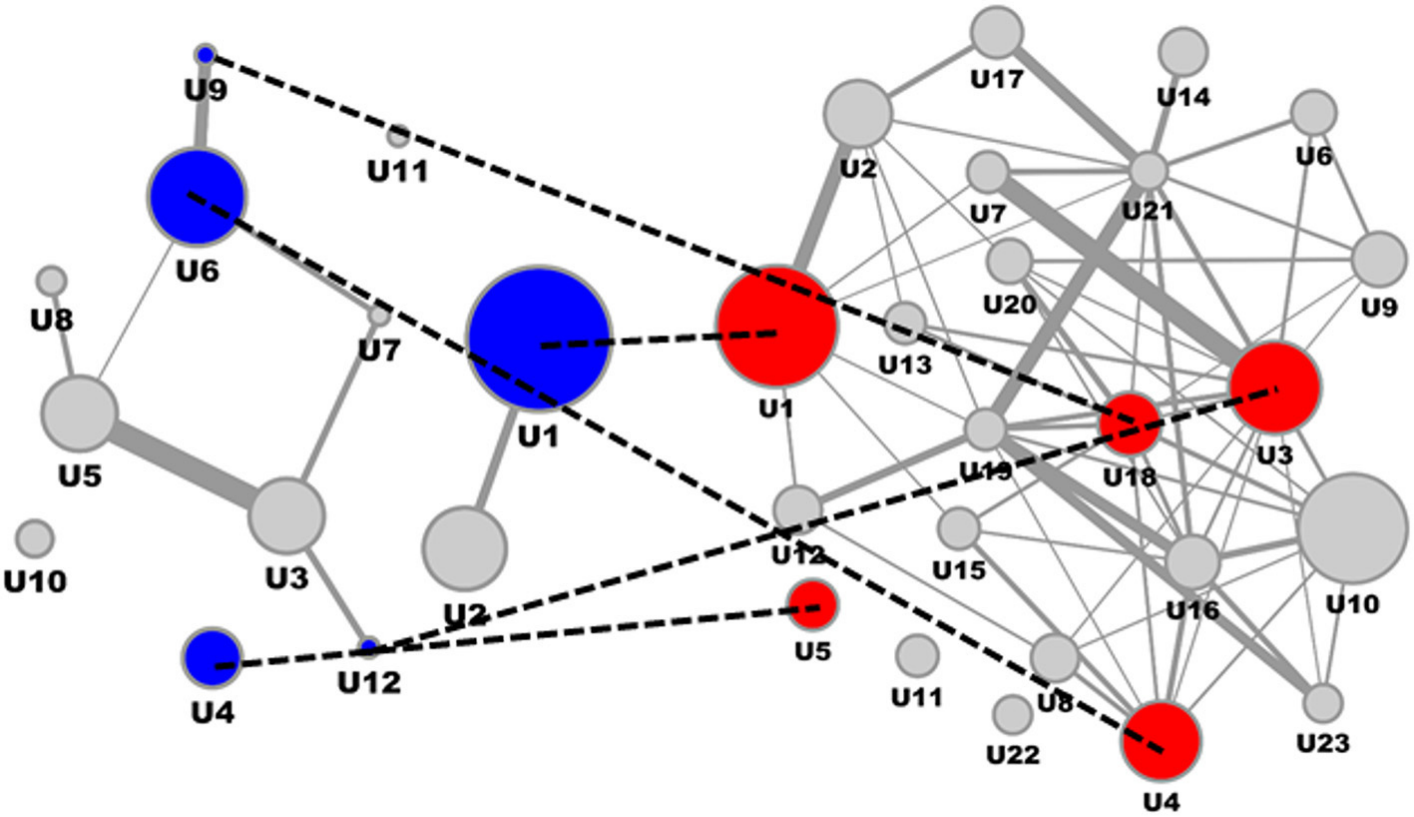
**ACE inhibitor mechanism of action molecular model and interference with DN molecular model**. ACE Mechanism of Action molecular model (left) and DN molecular model (right), with overlapping process segments of drug and phenotype models indicated by dotted lines. Molecular process segments (U) of the ACE mechanism of action molecular model showing interference with the DN molecular model are given in blue, respective interacting process segments on the DN side are given in red.

Consolidating transcriptomics signatures reflecting the impact of ACE inhibitors on the kidney interactome in a rat model utilizing six representative drugs resulted in 2058 molecular features (Table 2), with 661 features being identified in a least two of the six drug signatures. Mapping this consensus ACE feature subset on the consolidated interaction network allowed representation of 656 features. The induced subgraph included 332 features, after segmentation resulting in 12 process segments holding in total 92 molecular features (Figure 3, left). Median process feature set size was 8, with a maximum of 19 and a minimum of 3.

Interfering the ACE mechanism of action molecular model with the DN molecular model on the level of feature overlap (Figure 3) identified specific process segments of the DN molecular model also holding biomarker candidates (Table 4).

**Table 4:** **Diabetic nephropathy process segment interference**.

**Segment**	**# Genes in segment**	**Interference overlap**	**Biomarker candidates**	**Enriched pathways**
1	29	7	CCL5	Chemokine signaling; Cytokine-cytokine receptor interaction; Renin-angiotensin system; Complement and coagulation cascades
18	11	2	HBA1, NFKB1, HP, HBA2	–
3	20	3	TGFB1	ECM-receptor interaction; TGF-beta signaling; PI3K-Akt signaling
4	16	2	ACTA1	–

*Provided is the process segment number of the DN molecular model, number of genes assigned to the segment, number of features identified as affected according to the drug mechanism of action model, biomarkers involved in the segment (gene symbols), and relevant pathways from the DN pathway map being enriched in such segment*.

All four process segments of DN showing interference with the ACE drug mechanism of action model hold biomarker candidates. Two segments provide significant enrichment also on the level of molecular pathways, showing an integration of chemokine and cytokine signaling, RAS and complement and coagulation cascades for one process segment, the second process segment reflects components of PI3K-Akt signaling in the context of TGFB signaling and ECM receptor interaction.

Biomarker candidates serving as proxy for the interference of ACE and DN molecular models involve the chemokine (C-C motif) ligand 5 involved in immunoregulators and inflammatory processes, hemoglobin alpha 1 and 2 together with haptoglobin, the cytokine transforming growth factor, beta 1, along with the transcription factor NFKB1, finally including actin, alpha 1 involved in cell motility, structure and integrity.

## Discussion

For a large spectrum of clinical presentations an impressive number of drug targets have been proposed out of translational and preclinical research, with a significant number further proceeding into clinical trials. Just in the first half of 2014 close to 10,000 new clinical studies were recorded on the platform clinicaltrials.gov. Taking a specific look at diabetic nephropathy as clinical phenotype, 124 interventional trials in any status are identified at clinicaltrials.gov specifically involving the disease term, covering 45 individual drug entities addressing 86 known targets. Via mining scientific literature additional 18 drug targets are identified.

Next to a number of trials utilizing drugs and drug combinations addressing known factors impacting DN progression as the RAS, drug targets are disparately distributed across molecular pathways, hence mechanisms assigned to the disease.

From literature mining 27 different pathways according to KEGG and Panther pathway annotation are discussed as associated with DN, of which 19 hold drug targets. These include well known mechanisms of relevance in DN including hypoxia response or fibrosis, combined with a large set of signaling components. On top, 52 drug targets are embedded in molecular context outside this literature-derived DN pathway landscape.

For biomarker candidates an equivalent situation is found. 54 unique proteins extracted from scientific literature are discussed in any biomarker context, covering 14 of the 27 pathways, with 31 biomarker candidates not assigned to any of the members of the extracted DN pathway map.

Interestingly, predictive performance regarding disease progression of any of the individual biomarker candidates proved limited value. For example, in a review by Hellemons et al. 13 relevant markers were found in the context of nephropathy in diabetes, of which five were found as significantly associated with onset as well as progression of DN again covering various mechanisms including inflammation (e.g., C-reactive protein), cell surface interaction and homeostasis (e.g., E-selectin, ICAM1) and metabolism (triglyceride levels) (Hellemons et al., 2012).

Apparently, individual biomarkers reflecting the status of an individual molecular process, pathway or mechanism cannot capture disease prognosis for the comprehensive DN population. In alternative approaches multimarker panels were included in classifiers on disease diagnosis and prognosis demonstrating improved performance also in blinded validation. In Roscioni et al. a signature of 273 peptides determined in urine were included in a support vector machine-based classifier (Roscioni et al., 2013). The signature held fragments of collagen eventually mirroring alterations in the extracellular matrix turnover and fibrosis together with markers of inflammation as e.g., the pro-inflammatory protein S100-A9, as well as uromodulin shown to be associated with interstitial fibrosis and tubular atrophy (Nkuipou-Kenfack et al., 2014).

One contributing factor for needing multimarker panels may be individual variance of baseline biomarker levels, where inclusion of multiple markers specifically in non-linear classification methods adds to robustness. However, a second factor may be generic heterogeneity of the patient population. Specific disease presentation may significantly vary not only across stages of disease progression eventually seeing a transition from protective to damaging mechanisms, but even within a specific chronic kidney disease category as defined by present clinical classification provided by KDIGO guidelines (KDIGO Board Members, 2013).

Improved prognostic performance of multimarker panels on top of strict functional classification of stage transitions in DN utilizing albuminuria but also eGFR as clinically used progression parameters clearly support the case of pathophysiological heterogeneity of a, in present clinical terms homogeneous, patient population. However, specifically for albuminuria the role of functional marker vs. factor in disease is discussed (Roscioni et al., 2014).

Deriving robust diagnostic or prognostic classifiers from e.g., proteomics or metabolomics profiling may add to clinical patient management regarding onset as well as intensity of therapeutic measures (Roscioni et al., 2013; Pena et al., 2014). Also in clinical trial design such enrichment strategies may be utilized by e.g., identifying individuals prone to fast disease progression, and randomizing in this high risk cohort into medication and placebo arm (e.g., Priority trial, clinicaltrials.gov reference NCT02040441).

Prognostic biomarkers in contrast to diagnostic parameters with known assignment to molecular processes and pathways further allow an approximation of what specific mechanisms are associated with disease progression. The DN pathway landscape discussed in this work is solely a cross-sectional representation of the disease, in a first place not allowing deciphering which of the 27 individual pathways drive disease progression, and which other pathways are just bystanders or downstream consequences of mechanistic factors of disease. Hence, evaluating biomarker candidates for their association with progressive disease in turn allows determining mechanisms associated with progressive disease. Such knowledge is vital e.g., for determining novel drug targets, demanding to be embedded in disease mechanisms being factors for progressive disease. Remaining question however is if such mechanisms are relevant to the same extent or at all for a specific patient assigned to a clinical phenotype.

A prognostic biomarker set covering all potentially relevant processes enables specific molecular phenotyping of individual patients, being however not sufficient in terms of predicting drug response as a drug mechanism of action is not factored in. Here Systems Pharmacology aims at identifying drug response also on the level of molecular processes and pathways. Rationale is to not only focus on the specific drug target and its assignment to specific mechanisms, but to include the systemic molecular changes triggered by the drug including off-target effects as well as downstream molecular changes. Having a drug mechanism of action as well as a clinical phenotype represented on a molecular process or pathway level allows intersecting both molecular states. If from prognostic biomarker profiling of a patient specific progression-associated molecular disease mechanisms are identified, and a drug exhibits functional interference in such specific mechanisms such patient may be more prone for showing response to the drug. With such setting including knowledge on molecular phenotype composition, molecular process relevance in progressive disease and knowledge on interference of drug mechanism of action biomarker candidates initially serving a prognostic purpose can be rendered into predictive biomarkers on drug response.

Omics profiling has a major contribution to characterizing both, clinical phenotypes as well as drug mechanism of action. Integrating profiling results from clinical samples frequently sees minor overlap of individual studies, being in part driven by insufficient sample size combined with diverging inclusion criteria and sample material used (Fechete et al., 2011). In the example presented here 1010 features in total are identified as differentially regulated in transcriptomics or are being assigned to DN according to literature mining, with 880 unique features. An equivalent misbalance in feature coherence across studies is also found for the ACE inhibitor transcriptomics data. All these drugs address the same functional context, but from the in total 3152 features identified for six drugs included the total number of unique features are still 2058, with 661 being identified in at least two drug signatures.

Next divergence becoming apparent is the limited overlap of enrichment analysis based on signatures from profiling and feature-based literature mining compared to explicit literature mining for molecular pathways. Of the 27 pathways extracted from scientific references only seven are confirmed, however, seeing other pathways enriched not found via literature mining. On top, a major shortcoming is restricted representation of protein coding genes in such pathway maps, e.g., for KEGG covering 6491 and for Panther 2163 protein coding genes, respectively. This limitation not only affects pathway enrichment but also assignment of biomarker and target candidates. Of the in total 104 drug target and 54 biomarker candidates 29 are neither assigned in any KEGG or Panther pathway.

Here a different approach may be followed, namely segmentation of protein interaction networks exhibiting improved coverage of the protein coding gene set. Consolidation of INTACT, Reactome, and BioGRID allows representation of in total 13,907 protein coding genes, clearly expanding beyond public domain pathway databases. In alternative approaches hybrid interaction networks are utilized for further expanding coverage of protein coding genes, but also for improving false negative rates regarding protein-protein interactions and relations (Fechete et al., 2013).

Computing a DN-specific as well as ACE inhibitor-specific induced subgraph followed by topology-based segmentation allows an alternative representation of a molecular process landscape for the clinical presentation as well as the drug mechanism of action. Interference analysis on the level of overlapping protein coding genes resulted in four process segments holding central aspects of DN pathophysiology. Seven biomarker candidates were identified in these interfering molecular processes. CCL5 (RANTES), involved in recruiting monocytes and macrophages to the renal cortex was shown to be suppressed by ACE inhibition, indicating that RANTES expression is mediated via Angiotensin II type 2 receptor (Kashiwagi et al., 2002). Equivalently, in animal models TGFB1 expression was shown to be reduced by ACE inhibitors. Activation of NFKB1 by angiotensin II was shown in vascular smooth muscle and mesangial cells (Hernández-Presa et al., 1997). In a study by Dong et al. analyzing cost effectiveness of ACE inhibitor treatment for patients with type 1 diabetes mellitus the level of glycosylated HbA1c showed clear impact on cost effectiveness of drug use per quality-adjusted life year (QALY) (Dong et al., 2004). The authors concluded that next to patient age also other factors need to be included in therapy considerations.

Apparently, drug mechanism of action affects numerous molecular processes, as exemplified for ACE inhibitors, many of these also afflicted with DN progression. Analyzing the molecular process interface of disease progression-relevant pathophysiology and drug mechanism of action allows proposing predictive markers. Testing such predictive biomarker candidates may educate on relevance of individual processes on a patient level, directly linking to likelihood of drug response.

### Conflict of interest statement

The authors declare that the research was conducted in the absence of any commercial or financial relationships that could be construed as a potential conflict of interest.
